# Unraveling the intricate relationship: Influence of microbiome on the host immune system in carcinogenesis

**DOI:** 10.1002/cnr2.1892

**Published:** 2023-09-14

**Authors:** Saksham Garg, Nikita Sharma, Bharmjeet, Asmita Das

**Affiliations:** ^1^ Department of Biotechnology Delhi Technological University Delhi India

**Keywords:** cancer, dysbiosis, gut microbiome, immunotherapy, tumor microenvironment

## Abstract

**Background:**

Cancer is an outcome of various disrupted or dysregulated metabolic processes like apoptosis, growth, and self‐cell transformation. Human anatomy harbors trillions of microbes, and these microbes actively influence all kinds of human metabolic activities, including the human immune response. The immune system which inherently acts as a sentinel against microbes, curiously tolerates and even maintains a distinct normal microflora in our body. This emphasizes the evolutionarily significant role of microbiota in shaping our adaptive immune system and even potentiating its function in chronic ailments like cancers. Microbes interact with the host immune cells and play a part in cancer progression or regression by modulating immune cells, producing immunosuppressants, virulence factors, and genotoxins.

**Recent Findings:**

An expanding plethora of studies suggest and support the evidence of microbiome impacting cancer etiology. Several studies also indicate that the microbiome can supplement various cancer therapies, increasing their efficacy. The present review discusses the relationship between bacterial and viral microbiota with cancer, discussing different carcinogenic mechanisms influenced by prokaryotes with special emphasis on their immunomodulatory axis. It also elucidates the potential of the microbiome in transforming the efficacy of immunotherapeutic treatments.

**Conclusion:**

This review offers a thorough overview of the complex interaction between the human immune system and the microbiome and its impact on the development of cancer. The microbiome affects the immune responses as well as progression of tumor transformation, hence microbiome‐based therapies can vastly improve the effectiveness of cancer immunotherapies. Individual variations of the microbiome and its dynamic variability in every individual impacts the immune modulation and cancer progression. Therefore, further research is required to understand these underlying processes in detail, so as to design better microbiome‐immune system axis in the treatment of cancer.

## 1 INTRODUCTION

In the last couple of decades, our apprehension of the microbial world has increased significantly. The understanding of microbes especially those associated with the human body became important as several studies suggested the microbes being associated with numerous metabolic activities, including healthy and stress‐induced conditions. The microbiota is such a crucial component of the human body that it is seldom referred to as a “forgotten organ”.^1^ An estimate of a trillion microbes inhabits human anatomy and our comprehensive understanding and appreciation of the prokaryotic kingdom are attributed to the revolutionizing technologies over time. Recent evidence has proposed that an imbalance of the whole microbial ecosystem (dysbiosis) is responsible rather than a particular microorganism for various conditions. This disturbed microbiome is also recognized in fatal ailments such as cancer.^2^, ^3^

The role of both inflammatory reactions and metabolic products of microbes in cancer was soon acknowledged and was found to be tumor‐promoting as well as tumor‐suppressing.^4^, ^5^ Although the role of microbes in cancer was hypothesized more than a century ago by Virchow,^6^ ongoing research demonstrated that about 18% of all cancers in the world are related to infectious agents.^7^, ^8^ *Helicobacter pylori*, a common inhabitant of the gut microbiome, is officially given the status of class I carcinogen by International Agency for Research on Cancer (IARC),^9^, ^10^ and many other microbes and viruses are under scrutiny for their connection with different types of cancers.^11^, ^12^, ^13^, ^14^ The relationship is very complex and difficult between cancer and microbes. Generally, cancer is considered to be a disease of host genetics and environmental factors. The microbiota present in the human gut helps in the detoxification of dietary components, reducing inflammation, and maintaining balance in proliferation and host cell growth.^15^ However, scientists have given evidence to suggest a strong connection between microbial infections and cancer. The tumor can develop due to inflammatory responses that are induced by bacteria, secretion of bacterial toxins and enzymes, and oncogenic peptides.^16^ The present review discusses the fine balance between the microbiome of the body and the triggering of carcinogenesis and also the unique mechanism by which the modulation of the immune system by the microbiome can be cleverly used as a combinatorial therapeutic mechanism for cancer.

## 2 MECHANISMS CONTROLLING THE HOST INTERACTION WITH MICROBIOTA INVOLVED IN CARCINOGENESIS

The human body harbors different microbial species that have their own set of relationships such as commensalism, parasitism, and mutualism.^17^, ^18^ The human gut is a major habitat for an infinite number of bacterial strains and species, showing a symbiotic relationship between humans and microbes. An anatomical barrier that is, primarily the epithelial lining is essential to carry out the smooth functioning of this symbiotic relationship. The multi‐level barrier ensures this separation, and perforation or disruption of this barrier can lead to several diseases and also cancer. In addition to barrier protection, other additional physiological features like mucous layer, low pH, and stratum corneum also contribute to either maintenance of the barrier or directly shaping the microbial community inside the host. The host has also developed some special cells, such as goblet cells, paneth, and keratinocytes, which monitor the bacterial community and actively participate in shaping the microbial population by secreting antibacterial peptides.^19^, ^20^ Elements of the immune system are also a part of the barrier system for protection. The invading bacteria also respond in order to survive and protect themselves in the hostile environment. The mucin layer and bacteriocins production are the main mechanisms that bacteria implement to proliferate in a hostile environment.^21^ In the competition for resources, bacteria produce bacteriocins, which in turn are helpful for the human too as bacteriocins suppress the pathobionts, thus limiting the pathogenicity of the microbes.^22^ A supportive narrative is given by the fact that germ‐free mice, showed increased susceptibility to the invading bacteria and infections.^23^

As is often observed, the microbiota‐host interaction is hanging by a string of equilibrium, the disturbance in this equilibrated system is the cause that makes micro‐organisms cause disease. The failure is analogous to the domino effect; the defective working of even one mechanism (including both host and microbial) is followed by a defect in another mechanism. With all the dominos falling, overall equilibrium is disrupted, which is the essential cause for many diseases and often carcinogenesis too. *H. pylori* is a good example of the domino effect as its infection of the host harms not only the barrier cells but also causes increased inflammation, disrupts the microbiota causing dysbiosis, and also changes the gastric environment.^24^ The relationships between certain microorganisms and different kinds of cancer are shown in Table 1. This table emphasizes the microbial dysbiosis seen in many tumors and offers details on the possible involvement of these bacteria in the development of cancer.

**TABLE 1:** Various microbes involved in different types of cancers.

S. No.	Type of cancer	Influencing pathogen	Mode of action	References
1.	Lung cancer	*Bacillus* sp.	Chronic inflammation and the generation of metabolites that can lead to cancer	^25^
		*Mycoplasma* sp.	Triggering persistent inflammation, altering immunological responses, and perhaps having an impact on carcinogenic pathways	
		*Staphylococcus epidermis*	Indirectly influence immunological responses and the general makeup of the lung microbiota, which in turn may lead to lung cancer.	
		*Capnocytophaga*	By aspirating oral infections into the lungs and causing subsequent chronic inflammation	^26^
		*Veillonella*	Associated with respiratory infections	
		*Klebsiella*	Exacerbate tumor growth, compromise immunological function, and cause persistent inflammation	^27^, ^28^
		*Moraxella catarrhalis*	Having it in the respiratory system may cause persistent inflammation and encourage the growth of lung cancer	
2.	Pancreatic cancer	*H. pylori*	Via oncogenic signaling cascade activation, persistent inflammation, and host immune response modification	^29^, ^30^, ^31^
		*Pseudomonas aeruginosa*	Through the generation of virulence factors, activation of chronic inflammation, and possibly disruption of anticancer immune responses	^32^
		*Fusobacterium* species	Provoke chronic inflammation, modify the tumor microenvironment, encourage cell invasion, and affect immune responses	^33^
		*Streptococcus mitis*	Encouraging immunological dysregulation, and inflammation	^34^, ^35^
		*Porphyromonas gingivalis*	Cause prolonged inflammation, and interfere with the immunological response	
		*Neisseria elongate*	Immunological dysregulation and persistent inflammation.	^36^
3.	Gall bladder cancer	*Salmonella typhi*	Chronic inflammatory response resulting in DNA damage and cell growth	^37^
		*Helicobacter pylori*	Oncogenic signaling pathways and chronic inflammation.	^38^
4.	Ovarian cancer	*Mycoplasma* sp.	Immune control, long‐term inflammation, and direct contact with ovarian cells	^39^
		*Chlamydia pneumonia*	Immune control, long‐term inflammation, and direct contact with ovarian cells	^40^
5.	T‐cell leukemia	Human T‐cell leukemia virus‐1	DNA insertion into the host cell, cellular function impairment, and cell division	^41^, ^42^, ^43^
6.	Nasopharyngeal cancer	Epstein–Barr virus	Epithelial cell infection, transformation, and immune response evasion	
7.	Kaposi sarcoma	Kaposi sarcoma‐associated herpesvirus	Immune and endothelial cell infection, as well as growth factor and pro‐inflammatory cytokine release	
8.	Merkel cell carcinoma	Simian virus 40	DNA insertion into the host cell, interference with cellular control, impairment of cell growth and survival	
		Merkel cell polyomavirus	DNA insertion into the host cell, interference with cell cycle regulation, and cellular transformation	
9.	Skin cancer	Merkel cell polyomavirus	Cellular transformation, viral oncogene expression, and integration into the DNA of the host cell	^44^

## 3 MICROBIOME INFLUENCING HOST IMMUNE RESPONSE

For the survival of the individual organism or species, developing defense mechanisms against diseases is a crucial part of evolution. The crosstalk between microbiome and human cells plays a vital role in the development of both innate and acquired immunity.^45^ It is estimated that a human host trillions of microbes at various sites in the body.^46^ This results in extensive interactions and the immune system develops itself to attack the harmful microorganisms.^47^

Right after birth, the immune system begins to take shape, and it continues to mature over the course of a person's lifespan. In just a few weeks after birth, the developing newborn serves as the host to the whole microbial population. The research indicated that nursing on mother's milk is a crucial source for gut microbiota colonization in a newborn, while it is unclear exactly what should characterize the initial encounter with the microorganisms.^48^ Specific microbial strains like *Lactobacillus*, *Enterococcus*, *Bifidobacterium*, and *Staphylococcus*, are commonly found in breast milk and infant stool samples.^49^, ^50^ Moreover, the similarity of bacterial strains in the stool sample has been shown to be increasing proportionately with an increased intake of breast milk.^51^ These studies suggested a clear transfer of microbes from breast milk to the infant's gut, which is inevitable and dynamic. After initial exposure to the prokaryotic kingdom, the interaction and interplay increase with time thus, paving the path for development and increasing the tolerance of the human immune system. Important immune‐modulating effects of the microbiome on cancer development are shown in Table 2.

**TABLE 2:** Key immune‐modulating effects of the microbiome.

Immune modulation	Mechanism
Tumor immune surveillance	APC activation and T cell reactions
Regulatory T cell function	Inhibition of regulatory T cell induction
Inflammation	Modulation of inflammatory signals both pro and anti
Immune checkpoint regulation	Influence on the expression and activation of immunological checkpoints
Response to immunotherapy	The effectiveness and responsiveness to immunotherapies are affected

With the discovery of pattern‐recognition receptors (PRRs) and our growing understanding of the complex interaction of immune cells with the microbial community, humans have realized that despite the capability of our immune system to selectively destroy all prokaryotic microbes evolution has taught our immune system to tolerate the harboring of the normal flora. The PRR superset consists of a subset‐families with known characteristic pathways for each subset including Toll‐like receptor (TLRs), nucleotide‐binding oligomerization (NOD)‐like receptor (NLRs), the RIG‐I‐like receptor, the C‐type lectin receptor, the absent in melanoma 2 (AIMS2)‐like receptors, and the OAS‐like receptors.^52^ These receptors produce a specific response to an active stimulus. This sensing system of multiple levels and components has an analogy with a rheostat; according to the processed information at different levels of physiology, the appropriate adjustments are made to fit the host with the neighboring microbial ecosystem.^45^ At times, disruption in this intricate balance between humans and microbes is observed and called dysbiosis. In a nutshell, dysbiosis is an unstable microbiota that can lead to opportunistic infections.^53^ External agents such as probiotics are often supplemented for the re‐establishment of beneficial microbes or eubiosis, which strengthens the immune responses to treat dysbiosis.^54^ Overall, the immune system can be considered as a buffer to metabolic activities and foreign interactions as it configures both the response against and for the proliferation of microbial community by promoting the growth of microbiota that is helpful to the host and simultaneously shaping the existing microbiota, which is tolerable by the host.

TLRs are a component of the innate immune system and can produce high‐intensity pro‐inflammatory stimuli,^55^ depending on the pathway taken by the TLRs and the bacterial cell MAMPs they recognize, can either promote or protect against carcinogenesis. While TLR2 promotes gastric cancer,^56^ lipopolysaccharide and TLR4, increase colorectal, liver, pancreatic, and skin cancers.^57^, ^58^, ^59^, ^60^ TLRs are connected to cancer formation and survival, but it is not apparent if a direct or indirect role is being played. In order to promote pro‐carcinogenic pathways, NF‐κB and STAT3 are activated,^56^, ^57^, ^61^ and mitogens such as amphiregulin, epiregulin, and hepatocyte growth factor (HGF) produced by TLR‐expressing fibroblast cells have cancer‐causing effects.^57^, ^61^, ^62^, ^63^ Microflora‐induced TLR activation on myeloid cells results in the activation of cancer‐causing pathways such as IL‐17 and IL‐23. The carcinogenic nature of these cytokine networks is confirmed by the evidence that decreased IL‐17 and IL‐23 expression either by genetic suppression of *Tlr2*, *Tlr4*, *Myd88*, or *Tlr9* or rooting out the microflora by antibiotics, resulted in the reduced propensity for carcinogenesis in tissues.^64^, ^65^ As demonstrated by the case of MYD88, where MYD88 inactivation suppresses cancer but may potentially result in colorectal cancer (CRC) linked to the damaged epithelium, TLRs also play a protective function.^59^, ^66^ Through the downregulation of pro‐inflammatory IL‐22 and promotion of epithelial damage repair, the protective IL‐18 pathway, which is controlled by the MYD88 protein, confers a protective character to the colonic epithelium. The development of colitis‐associated cancer (CAC) is shown in IL‐18‐deficient mouse models, which is equivalent to the outcomes of Myd88‐/‐mice models.^67^ Therefore, the absence of an IL‐18 pathway might be the cause of colon cancer. A subgroup of the PRR superfamily called NOD‐like receptors (NLRs) has a NACHT domain situated at the center, which is crucial for oligomerization and leucine‐rich repeats are present toward C‐terminal facilitating ligand identification.^68^, ^69^ A group of NLRs known as NLRP has a pyrin domain (PYD) out of which NLRP6 has been extensively studied in host‐microbiome interaction and is best known for inducing inflammasomes.^70^ and contributes to immunity against bacteria, which is represented by dysbiosis in *Nlrp6* ^−/−^ mice, making them more prone to colitis and CRC progression.^71^, ^72^, ^73^ The proposed molecular mechanism in *Nlrp6* deficit mice is the reduced stimulus to inflammasomes and IL‐18 production. Greater susceptibility to CRC is shown by *Asc* ^−/−^and IL‐18 deficient mice confirming the proposed mechanism.^70^ A muramyl dipeptide‐sensing NLR called NOD2 aids in preserving immunity against microorganisms.^74^ Dysbiosis brought on by mutations in NLRP6 and NOD2 can result in CRC.^75^, ^76^ umari et al showed that the intestine of *Nod2* ^−/−^ mice model is usually found in a state of dysbiotic distress.^77^ All these pieces of evidence shift the belief that the involvement of NOD2 in cancer is via dysbiosis as this increased cancer propensity was seen to be transferable too in co‐housing.^76^ Both NOD2 and NLRP6 share IL‐6^78^ as a common mediator, and treatment with IL‐6‐neutralizing antibodies to knockout models reduces the development of CRC.^70^, ^76^ By promoting inflammation and inducing CRC, NOD1 deficiency also influences carcinogenesis. More research is required to determine the precise mechanism behind the carcinogenic impact of other NLR family members.

## 4 INFLUENCE OF MICROBIOTA ON CARCINOGENESIS THROUGH VIRULENCE FACTORS AND GENOTOXINS

**FIGURE 1:**
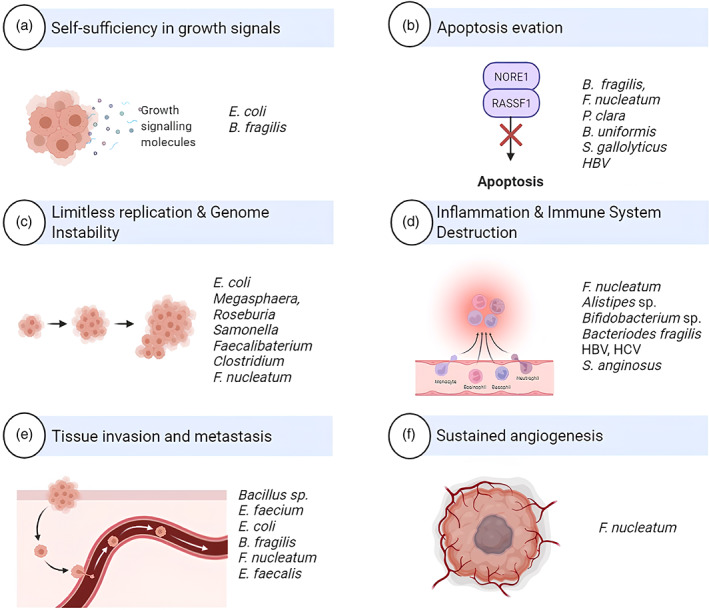
Microorganisms at different stages of cancer.

Microbes are extremely important for the development, progression, and even response to therapy of cancer. New research reveals that the human microbiome, which consists of a wide variety of bacteria living in and on our bodies, might affect the development and course of cancer. At the beginning of the disease, some bacteria can directly cause DNA damage or encourage persistent inflammation, fostering conditions that allow healthy cells to develop into malignant cells. Through a variety of processes, including immune system regulation, modification of the tumor microenvironment, and creation of chemicals that support tumor cell survival and proliferation, the microbiome can influence tumor development, invasion, and metastasis as cancer progresses. Furthermore, the microbiome can change the way drugs are metabolized, regulate immune responses, and possibly affect how well immunotherapy works. Figure 1 emphasizes how bacteria play a role in the initiation of DNA damage, the promotion of chronic inflammation, the influence on the growth and spread of tumors, and the modulation of therapeutic responses.

Virulence factors are transcribed products that mediate the establishment of a pathogen in the host cell, increasing its pathogenicity. These may include bacterial toxins, hydrolytic enzymes, surface protein, and carbohydrates.^79^ Some factors such as vacuolating cytotoxin A (VacA), cytotoxin‐associated gene A (CagA), and FadA have been extensively researched for their potential to cause cancer.^6^ CagA and VacA are developmental agents of gastric cancer in mice models. FadA mediates the adhesion of bacterium to the host cell. However, FadA has been observed to interact with other compounds too, such as E‐cadherin activating β‐catenin signal leading to CRC.^80^, ^81^

Metabolism of xenobiotics is considered to play a crucial role in gut microflora.^82^ Such metabolism affects the activity and toxicity of the drugs and therapeutic compounds that are administered. One example is the case of irinotecan (CPT‐11), a drug used for the treatment of colon and pancreatic cancer.^83^ In the liver, the metabolism of irinotecan terminates by its inactivation, but in the presence of β‐glucuronidase, the compound is reactivated and thus contributes to additional severe side effects like diarrhea, which also limit the potency of the drug. The inactivation of bacterial enzymes or treatment with antibiotics are some of the measures that are taken to reduce these complications and increase the potency of the particular drug molecule.^84^

Microflora present in the intestine regulates bile metabolism using hydrolase enzymes as the medium of action. Deoxycholic acid (DCA) is one of the modulated bile salts. In a diet model with high‐fat content, DCA is an appurtenance to another Hepatocellular carcinoma (HCC) causing agents. Diet changes and reduction in DCA‐producing bacteria using antibiotics decrease the chances of developing HCC. DCA is being implicated in colon and esophageal cancers also,^85^, ^86^, ^87^ suggesting bacterial involvement in a multitude of cancers.

Around 3.6% of all cancers, including the rectum, pharynx, oral cavity, colon, female breast, esophagus, and liver, are implicated by alcohol, and the breakdown of alcohol to acetaldehyde is largely dependent on the microbiome present.^88^ Observations in germ‐free mice revealed a significantly lower concentration of acetaldehyde, thereby showing genotoxic effects and disease promotion. This conversion is also hypothesized to be crucial in oral cavity cancers as the concentration of acetaldehyde in the oral cavity is 10‐100 times higher than in blood vessels.^88^, ^89^

Additionally, DNA instability and genotoxicity caused by bacterial toxins and metabolic products can result in cancer.^90^ Bacterial toxins such as cytolethal distending toxin (CDT), colibactin, and cytotoxic necrotizing factors contribute to DNA malfunctioning and cytotoxicity disrupting the mediating cellular pathways. Bacteria‐induced inflammation is synchronized with reactive oxygen species (ROS) production, which also contributes to genotoxicity and has the potential to form tumors.^65^, ^91^, ^92^, ^93^, ^94^ A toxin produced by *Bacteroides fragilis* (*B. frgailis*) that causes bowel inflammatory diseases has been linked to CRC neoplasia and cancer.^95^, ^96^ Infection with *Salmonella* sp. has been reported to have a genotoxic effect by suppressing the adenomatous polyposis coli (APC) gene, a tumor suppressor gene thus, increasing the risk of generation and proliferation of cancer.^97^ Both CDT and colibactin toxins exert extensive DNA damage by ataxia‐telangiectasia mutated (ATM)‐CHK2 pathway and histone H2AX phosphorylation leading to seizing cell cycle and swollen cells.^6^

CDT genotoxin can cause various cancers, including CRC, gastric, and gallbladder.^98^ CDT is an AB toxin with three subunits as CdtA, CdtB, and CdtC. CdtB is the only subunit that enters the cell cytoplasm, from where the active subunit invades the nucleus and confers DNA damage.^92^ The damaging subunit is highly homologous to the DNase I site, and this homology results in reduced damage control of DNA by the normal functioning enzymes.^92^, ^99^ In nuclear factor‐κB (NF‐κB) lacking mice models, the CdtB mutant bacteria failed to induce any cell growth in the intestine. Administration of the same bacteria to IL10^−/−^ mice models also failed to show any signs of dysplasia. These studies concluded that CDT‐mediated DNase activity is essential for certain groups of bacteria to form tumors and can be potentially carcinogenic.^100^, ^101^

**FIGURE 2:**
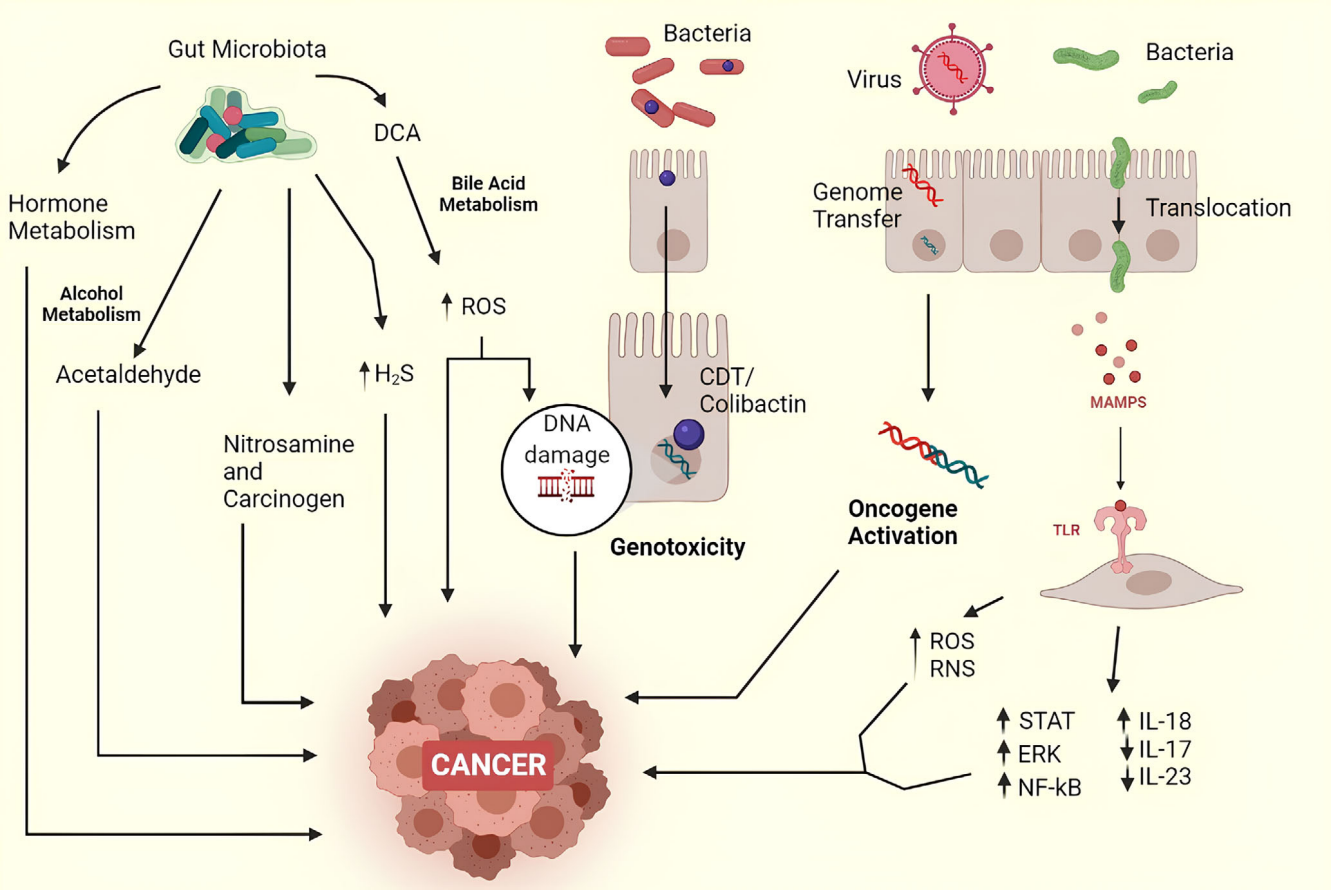
Microbial mechanisms in cancer progression.

Colibactin is encoded by 54 kb polyketide synthase (PKS).^102^ Some studies have linked the toxin with CRC development using the Il10^−/−^ mice model. *E. coli* with functionally active PKS islands have been isolated from the developing CRC tissues.^91^, ^103^ Colibactin is formed as a result of the inclusive activity of eight out of nine accessory genes and also includes non‐ribosomal peptide synthetase (NRPS). All these components are essential to produce functional colibactin. It is also hypothesized that carcinogenicity can be mediated by DNase. This hypothesis is supported by the study, which concluded that the structural integrity of DNA was affected in the cells having *pks* active bacterial strain.^102^ As toxin is rather newly observed, the exact direct relationship of the toxin needs further explanations and pieces of evidence. A thorough explanation of how microorganisms might contribute to cancer progression is given in Figure 2. The figure illustrates important microbiological pathways including persistent inflammation, genomic changes, immunological regulation, and synthesis of chemicals that support tumor growth.

At times, metabolic products released by the bacteria also contribute to genotoxicity and instability of DNA.^104^, ^105^ Prime examples of such cases are the production of hydrogen sulfide, superoxide compounds, and N‐nitroso compounds (NOC). Diet in such cases is a crucial point to consider as the gut microflora widely is dependent on the type of dietary components consumed.^106^ Sulfate‐reducing bacteria (SRB) are the cause of the H_2_S released in the gut, which is a genotoxic and cytotoxic gas.^104^, ^107^, ^108^ It damages the DNA as well as reduces ATP formation.^109^ SRB is a constituent part of gut microbiota. However, a diet with sulfur‐containing amino acids or sulfur‐polluted water can increase the concentration of SRB thereby, increasing the risk of CRC.^106^, ^110^ Superoxide radicals are observed to promote tumor formation in *Il10* ^−/−^ mice, specifically in the gut.^111^, ^112^ NOC shows alkylation of DNA, therefore, transitioning GC to AT causes major DNA damage in the K‐*ras* gene promoting CRC.^113^ Nitrogen‐reducing bacteria are again a part of normal gut microflora, but consumption of red meat diet increases the requirement of nitrogen‐reducing bacteria and, in turn, increases the metabolic NOC.^114^, ^115^ Detoxification and microflora management by control of diet and elimination process genotoxic compounds are likely to excrete out and accordingly affect carcinogenesis.

## 5 MICROBES AS PERSONALIZED CANCER TREATMENT

For the past 10 years, researchers have studied microbes as a therapy or in conjunction with other medicines. However, Dr. Coley first identified their application as anti‐cancer drugs in the 19th century, which is when their usage as cancer therapies began. The basis for the use of microorganisms in cancer therapy was laid by an experiment involving the injection of *Streptococcus pyogenes* in a patient with bone sarcoma that showed promising tumor shrinkage.^116^, ^117^ The use of microorganisms in the treatment of cancer is currently receiving fresh attention. Because various types of cancers exhibit heterogeneity and patients respond differently to therapies, precision medicine is an attractive cure for cancer. Table 3 shows that a number of clinical trials have looked at the potential of microbiome‐based therapies in the treatment of cancer. The research looked at several treatments, including fecal microbiota transplantation (FMT) for colorectal cancer, probiotic supplements for breast cancer, and the effect of antibiotic medication on the effectiveness of immunotherapy for lung cancer. By influencing the immune system, particularly inflammation pathways, microbe‐wide genomic investigations have demonstrated the significance of bacteria in both well‐being and disease. Despite the challenges involved in the process, recognizing this effect is essential for improving outcomes, and identifying targets, biomarkers as well as diagnostics.^118^

**TABLE 3:** Clinical trials examining cancer treatments based on the microbiome.

Cancer type	Intervention	Study design	Findings
Colorectal cancer	FMT	Randomized controlled trial	FMT decreased tumor development and increased survival.
Lung cancer	Antibiotic treatment	Retrospective cohort study	Use of antibiotics was linked to a decreased response to immunotherapy.
Breast cancer	Probiotic supplementation	Prospective interventional study	Probiotics increased the variety of bacteria in the stomach and decreased adverse effects from therapy.
Pancreatic cancer	Gut microbiota modulation	Randomized controlled trial	Patient survival and treatment responsiveness were both enhanced by gut microbiota modification.
Prostate cancer	Prebiotic supplementation	Single‐arm pilot study	Supplementing with prebiotics has the potential to alter the gut flora and lower inflammation.
Hepatocellular carcinoma	Microbiota targeted therapy	Pilot clinical trial	Therapy that targets the microbiota has the potential to enhance patient outcomes and liver function.
Ovarian cancer	Probiotic supplementation	Case–control study	Probiotics improved treatment outcomes

**FIGURE 3:**
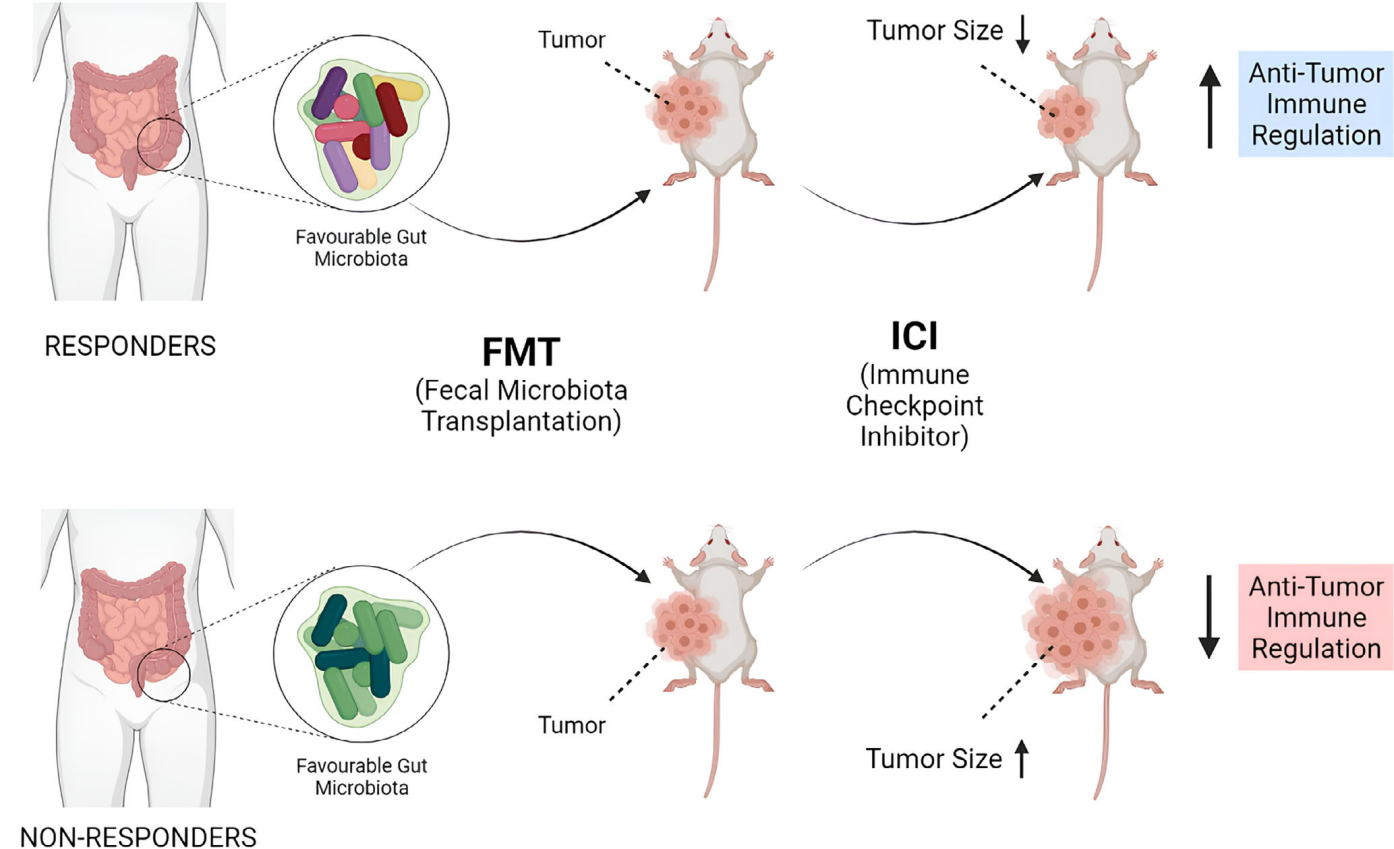
Microbial diversity and population present in the gut influence the regulation and response of immune checkpoint inhibitors (ICI).

The effectiveness of traditional cancer treatments has been enhanced with the use of combinatorial cancer medicines,^119^ and microbial therapies have been found to be effective when combined. Microbes and the immune system are intertwined, and they can affect how well immunotherapeutic treatments work. According to studies, the resident gut microbiota, which includes “favorable” and “unfavorable” microorganisms, might influence how differently people respond to the same medicine (Figure 3).

By eliciting pro‐inflammatory immune responses, *Bacteroides fragilis* and *Bacteroides thetaiotaomicron*, for instance, have been demonstrated to improve the effectiveness of CTLA‐4‐based blocking treatment.^120^ Fecal microbiota transplant FMT has been shown to increase anti‐tumor immunity and the overall effectiveness of PD‐L1 inhibition by reducing tumor development and boosting T‐cell responsiveness in non‐responding models.^121^ Patients with microbiota enriched in *Faecalibacterium* exhibited better results, which is indicative of the relative variety of microorganisms in the gut.^122^, ^123^ These results have been shown in solid tumors such as urothelial, renal cell, and lung carcinomas, where the presence of a lot of *Akkermansia muciniphila* favored a better prognosis.^124^

Since the microbial populations in the gut can enhance current treatments, such as immune checkpoint inhibition, they can be investigated for potential therapeutic benefits in cancer as well as other chronic illnesses. By identifying and modifying the gut microbiota, personalized medicine can be designed using sequencing and FMT that may improve cancer therapies augmentatively.

## 6 CONCLUSION

The complex interaction between the microbiome and the human immune system during carcinogenesis is now widely acknowledged as a key element in the onset and spread of cancer. Through a variety of processes, the microbiome affects the host immune system, having an impact on immune cell activity, immunological signaling pathways, and the tumor microenvironment. Dysbiosis and predominance of certain microbial communities have been linked to an increased risk of developing cancer, highlighting the significance of the makeup of the microbiome in the development of cancer.

Significant impacts on cancer prevention, diagnosis, and therapy may result from an understanding of the intricate interactions between the microbiota and the human immune system. An intriguing direction for future study is using the microbiome to modify immune responses and improve the efficiency of immunotherapies. Innovative treatment approaches to enhance patient outcomes can be created by using the microbiome‐immune system axis. However, there are a number of issues that must be resolved. Further research is necessary to fully understand the processes by which the microbiome affects immune responses and the development of cancer. Additionally, it is difficult to pinpoint specific microbial signatures linked to cancer because of the complexity of the microbiome makeup and the inter‐individual variability.

Nevertheless, the role of the microbiome‐immune system nexus in the treatment of cancer cannot be overstated. Future studies should concentrate on elucidating the complex processes behind this link, creating standardized microbiome analysis procedures, and carrying out clinical trials to evaluate the effectiveness of microbiome‐based treatments in preventing cancer and therapy. In conclusion, the impact of the microbiome on the human immune system in the development of cancer is an exciting field of study with broad ramifications. A changeable element that can be addressed for customized cancer treatments is the microbiome. Continued research in this area will surely improve our knowledge of cancer biology and open the door to cutting‐edge methods for treating this deadly condition.

## AUTHOR CONTRIBUTIONS

**Saksham Garg:** Data curation (lead); writing – original draft (lead). **Nikita Sharma:** Writing – original draft (supporting). **Bharmjeet:** Writing – original draft (equal). **Asmita Das:** Conceptualization (lead); project administration (lead); writing – review and editing (lead).

## CONFLICT OF INTEREST STATEMENT

The authors have stated explicitly that there are no conflicts of interest in connection with this article.

## ETHICS STATEMENT

The present work has been done in compliance with the ethical guidelines of Delhi Technological University.

## Data Availability

Data sharing is not applicable to this article as no new data were created or analyzed in this study.
